# The Trend of TIM3 Expression on T Cells in Patients With Nontuberculous Mycobacterial Lung Disease: From Immune Cell Dysfunction to Clinical Severity

**DOI:** 10.3389/fimmu.2021.738056

**Published:** 2021-11-10

**Authors:** Ping-Huai Wang, Ming-Fang Wu, Chi-Yu Hsu, Sheng-Wei Pan, Chin-Chung Shu, Shih-Lung Cheng

**Affiliations:** ^1^ Division of Pulmonology, Department of Internal Medicine, Far Eastern Memorial Hospital, New Taipei City, Taiwan; ^2^ Graduate Institute of Toxicology, College of Medicine, National Taiwan University, Taipei, Taiwan; ^3^ Institute of Statistical Sciences, Academia Sinica, Taipei, Taiwan; ^4^ College of Medicine, National Taiwan University, Taipei, Taiwan; ^5^ Department of Chest Medicine, Taipei Veterans General Hospital, Taipei, Taiwan; ^6^ School of Medicine, National Yang Ming Chiao Tung University, Taipei, Taiwan; ^7^ Department of Internal Medicine, National Taiwan University Hospital, Taipei, Taiwan; ^8^ Department of Chemical Engineering and Materials Science, Yuan Ze University, Taoyuan City, Taiwan

**Keywords:** apoptosis, cytokine, *Mycobacterium avium* complex, nontuberculous mycobacteria, lung disease, T-cell immunoglobulin and mucin domain-containing protein 3 (TIM3)

## Abstract

**Background:**

The incidence of nontuberculous mycobacterial lung disease (NTM-LD) is increasing worldwide. Immune exhaustion has been reported in NTM-LD, but T-cell immunoglobulin and mucin domain-containing protein 3 (TIM3), a co-inhibitory receptor on T cells, has been scarcely studied.

**Methods:**

Patients with NTM-LD and healthy controls were prospectively recruited from July 2014 to August 2019 at three tertiary referral centers in Taiwan. We examined TIM3 expression on the T cells from the participants using flow cytometry. TIM3 expression was analyzed for different disease statuses and after treatment. The apoptosis and cytokine profiles were analyzed according to the TIM3 expression.

**Results:**

Among enrolled subjects (47 patients and 46 controls), TIM3 on CD4+ cells (6.44% vs. 4.12%, *p* = 0.028) and CD8+ cells (18.47% vs. 9.13%, *p* = 0.003) were higher in NTM-LD patients than in the controls. The TIM3 level on CD4+ and CD8+ T cells was positively associated with T-cell apoptosis in the NTM-LD patients. In stimulating peripheral blood mononuclear cells using PMA plus ionomycin, a high TIM3 level on T cells correlated with low interleukin-2 and tumor necrosis factor-alpha (TNF-α) on CD4+ cells and interferon-gamma and TNF-α on CD8+ T cells. For clinical manifestation, low body mass index (BMI), positive sputum acid-fast smear, and high radiographic score correlated with high TIM3 expression on T cells. After NTM treatment, TIM3+ decreased significantly on CD4+ and CD8+ T cells.

**Conclusions:**

In patients with NTM-LD, TIM3+ expression increased over CD4+ and CD8+ T cells and correlated with cell apoptosis and specific cytokine attenuation. Clinically, TIM3+ T cells increased in patients with low BMI, high disease extent, and high bacilli burden but decreased after treatment.

## Introduction

The incidence of nontuberculous mycobacteria lung disease (NTM-LD) has increased over the last two decades and become an important clinical issue (1, 2). However, the etiology remains unclear (3, 4). Among NTM-LD, *Mycobacterium avium* complex (MAC) and *Mycobacterium abscessus* (MAB) are the most predominant pathogens in North America and East Asia, and they are the two most frequently isolated species responsible for NTM-LD (4, 5). Since MAC and MAB exist ubiquitously in the environment, airway colonization with MAC or MAB is not uncommon. In fact, less than half of patients with positive sputum cultures for MAC and MAB clinically have the disease (6, 7), indicating the importance of host vulnerability to NTM pulmonary infection (8).

Patients with NTM-LD become immunocompromised through complex host–pathogen interactions. Attenuated immune response of peripheral blood mononuclear cells (PBMCs) has been reported by MAC stimulation (9, 10). However, the pathogenesis of the immune attenuation in NTM-LD has yet to be understood. Our previous report showed that programmed death-1 (PD-1), a suppressive co-receptor for T-cell activation (11), was increased on lymphocytes in patients with MAC-LD and might play an essential role in attenuating host immunity (10). However, the decreased cytokine production has not been totally reverted by *in vitro* PD-1 blockade (10). In regard to other immune regulators, T-cell immunoglobulin and mucin domain-containing protein 3 (TIM3), a negative regulator of T helper 1 immunity, is similar to other T-cell inhibitory receptors, such as PD-1 (12, 13). Increasing expression of TIM3 on T cells may mediate the decrease of secretion of TNF-α and IFN-γ in viral infections, mycobacterial infection, or tumors (14–16). The prognosis of double positivity of TIM3 (+) and PD-1 (+) seems worse (14, 17). However, the role of the TIM3 pathway in modulating T-cell immune responses has been scarcely studied in NTM-LD in terms of clinical manifestation and cellular function. It is important to understand the pathogenesis and immune modulation. Therefore, we conducted this study to investigate TIM3 expression in NTM-LD patients and its role in clinical and cellular effects.

## Methods

### Patient Enrollment

This prospective study was conducted at the National Taiwan University Hospital (NTUH) and Taipei Veterans General Hospital (TVGH) from July 2014 to August 2019 and at Far Eastern Memorial Hospital (FEMH) from January 2016 to August 2017. The Research Ethics Committees of the hospitals approved the study (NTUH IRB No. 201407079RIND, 201505069RINC, 201701014RINC and 201705087RINA; TVGH IRB No. 2014-09-008BC and FEMH IRB: 107162-E,109004-E). Adult patients (≥20 years old) with at least two sputum cultures positive for the same NTM were recruited for the NTM-LD group based on the guidelines from the American Thoracic Society (ATS) (1). We excluded patients with active tuberculosis and human immunodeficiency virus infection. We included healthy controls with sputum-negative results for NTM and negative findings of NTM-LD by chest radiographic images. All final enrolled participants provided written informed consent.

### Collection and Staining of Peripheral Blood Mononuclear Cells

We sampled patients’ peripheral blood into heparin-containing tubes and isolated the mononuclear cells immediately using Ficoll-Paque PLUS (GE Healthcare Life Sciences, Sweden). We then stained PBMCs for CD4, CD8, TIM3, Annexin V, and Sytox Orange. The expression was measured using flow cytometry (FACSVerse, BD Biosciences, USA). The lymphocyte population could be discriminated by forward scatter (FSC) and side scatter (SSC), and the subgroups of CD4^+^ and CD8^+^ T lymphocytes could be gated. We measured the expressions of TIM3, Annexin V, and Sytox Orange.

### Stimulation Assay

We cultured PBMCs in 48-well plates and added Phorbol 12-myristate 13-acetate (PMA) (50 ng/ml, TOCRIS, USA) plus ionomycin calcium salt (1 μM/ml, Sigma-Aldrich, USA) for 16 h. We added protein transport inhibitor (BD Bioscience, USA) to the co-culture 12 h before we stopped the stimulation. After coculturing, PBMCs were stained for CD4, CD8, interleukin-2 (IL-2), tumor necrosis factor-alpha (TNF-α), interferon-gamma (IFN-γ), and TIM3 expression. The cytokine and immune checkpoint expressions were measured by flow cytometry and were analyzed in BD FACSuite V software (BD, Biosciences, USA).

### Antibodies

The staining antibodies were anti-CD4-APC, anti-TIM3-PerCP-Cy5.5, anti-IFN-γ-PerCP, anti-IL-2-PE, and anti-TNF-α-FITC antibodies (Biolegend, USA), and anti-CD8-FITC (BD Biosciences, CA, USA).

### TIM3 Blocking Assay

Because previous studies showed that healthy controls had response to the NTM antigen (10, 18, 19) and we aimed to investigate the general effect of TIM3 functional blocking on apoptosis occurrence, we enrolled MAC-LD patients and the healthy controls for the assay. After we collected the blood sample and isolated PBMCs, we cultivated PBMCs in 24-well plates (5 × 10^5^ cells per well) and added heat-killed *M. avium* bacilli (10) with MOI of 10 for 24 h with or without pretreatment of antagonistic TIM3 (10 μg/ml for 1 h) (clone F38-2E2, eBiosciences, USA). Apoptosis in the cocultured PBMCs was defined by Annexin V and Sytox Orange and was measured by flow cytometry.

### Data Collection and Statistical Analysis

We recorded clinical data, radiographic findings and laboratory data at enrollment. The clinical data included age, sex, body mass index (BMI), and comorbidities. An expert interpreted chest imaging by radiographic score (20). We recorded grade of acid-fast bacilli staining (AFS), which ranged from 0 to 4+, and results of *Mycobacterium* cultures of the sputum samples. Positive AFS indicated a high burden of NTM bacilli (21). We then classified those with low BMI, positive AFS, and high radiographic score for analysis of the role of TIM3 in disease severity.

We used the Mann–Whitney *U* test and Fisher’s exact test for continuous and categorical variables, respectively. A *p* value < 0.05 indicated statistical significance in the univariate analysis. All analyses were conducted in SPSS version 19.0 (Chicago, IL).

## Results

### Participant Demographics for Baseline TIM3 Expression

We enrolled a total of 93 participants for baseline TIM3 examination, including 47 patients with MAC-LD and 46 controls. The patients with MAC-LD were similar in age (63.7 ± 12.9 years vs. 61.7 ± 1 6.0 years, *p* = 0.442), but the proportion of females was higher (66% vs. 41%, *p* = 0.017) and BMI was lower (21.0 ± 3.7 vs. 23.0 ± 3.6 kg/m^2^) as compared with the controls. Ever-smoking rate (10.6% vs. 15.9%, *p* = 0.718) and prior TB history (12.8% vs. 2.3%, *p* = 0.171) were not significantly different in the two groups. Among the MAC-LD patients, 48.8% (*n* = 22) had positive AFS sputum.

### The Baseline Level of TIM3 Expression on CD4+ and CD8+ Lymphocytes

**Figure 1:**
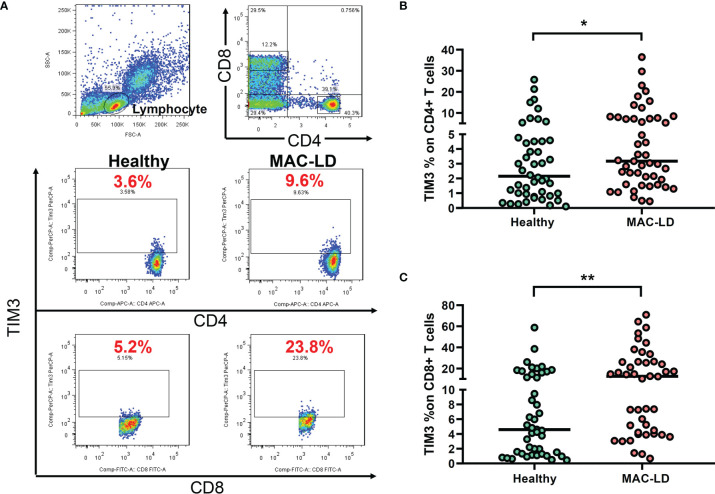
The expression of TIM3 on CD4+ and CD8+ lymphocytes according to disease status. **(A)** Case demonstration and **(B, C)** scatter plots between healthy controls and patients with *Mycobacterium avium* complex-lung disease (MAC-LD) are shown. The lymphocyte group was discriminated by forward scatter (FSC) and side scatter (SSC). CD4+ and CD8+ cells were then gated within the lymphocytes and TIM3 was measured. The cross bars in the scatter plots are median values. We compared the data by Mann–Whitney *U* test. One star indicates *p* < 0.05; and two stars mean *p* < 0.01.

As shown in **Figure 1A**, we gated lymphocytes and then CD4+ and CD8+, respectively, among the PBMCs. The expression of TIM3 was higher in CD4+ (6.44 ± 7.79% vs. 4.12 ± 5.52%, *p* = 0.028) and CD8+ T cells (18.47 ± 18.51% vs. 9.13 ± 11.41%, *p* = 0.003) in MAC-LD patients than in the controls (**Figures 1B, C**). Logistic regression showed that the percentage of TIM3+CD4+ (odds ratio [OR] 1.119, 95% CI: 1.012–1.237 per 1% increment, *p* = 0.029) and TIM3+CD8+ T cells (OR 1.071, 95% CI: 1.021–1.122 per 1% increment, *p* = 0.004) were correlated with MAC-LD in comparison with the controls. After adjustment by age and sex, the OR was still significant for TIM3+CD4+ cells (1.127 [1.012–1.254]) and TIM3+CD8+ cells (1.073 [1.020–1.128]).

### Apoptosis Associated With TIM3 Expression

**Figure 2:**
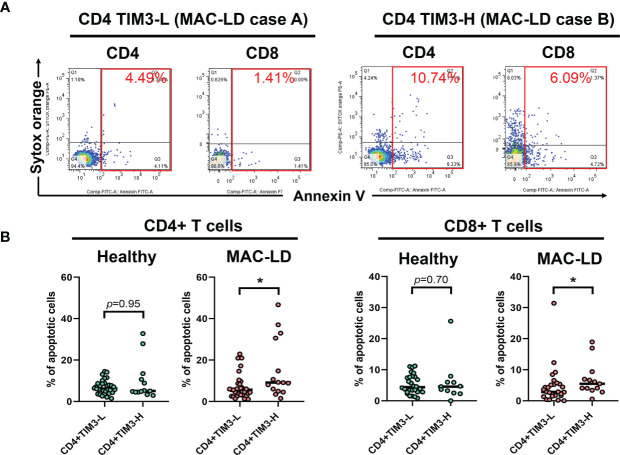
The expression of apoptosis percentages by CD4+TIM3-low (CD4+TIM3-L) or CD4+TIM3-high (CD4+TIM3-H) subgroups, defined by average TIM3+ level. The figure shows **(A)** case demonstrations for apoptosis staining (Annexin V and Sytox Orange) and **(B)** scatter plots comparing apoptosis percentage on CD4+ or CD8+ T cells between CD4+TIM3-H or TIM3-L subgroups among healthy controls or patients with *Mycobacterium avium* complex-lung disease (MAC-LD). Apoptosis % was the sum of early apoptosis [Anexin V+/Sytox Orange–] and late apoptosis [Annexin V+/Sytox Orange+]. The cross bars in the scatter plots are median values, and the significance was analyzed by the Mann–Whitney *U* test. One star indicates *p* < 0.05.

In measuring non-stimulation baseline PBMC, we classified TIM3+CD4+ expression as high (TIM3-H) and low (TIM3-L) based on the average level of 6.4% and used Annexin V and Sytox Orange to categorize apoptosis. **Figure 2A** shows that, compared to high TIM3+CD4+ level (TIM3-H, case B), TIM3-L (case A) was associated with declined apoptosis % (the sum of early apoptosis [Annexin V+/Sytox Orange–] and late apoptosis [Annexin V+/Sytox Orange+]). MAC-LD patients with high TIM3+CD4+ levels had significantly higher apoptosis (15.2 ± 14.2% vs. 7.1 ± 5.9%, *p* = 0.025) (**Figure 2**) on CD4+ lymphocytes than patients with low TIM3+CD4+ expression. In addition, significantly higher apoptosis (6.9 ± 6.4% vs. 4.7 ± 6.0%, *p* = 0.047) was found in CD8+ cells in MAC-LD patients with high TIM3+CD4+ expression. By contrast, there was no significant difference between the controls with high and low TIM3+ CD4+ expression. On the other hand, high and low expression of TIM3+ CD8+ did not have different apoptosis statuses in CD4+ and CD8+ cells.

**Figure 3:**
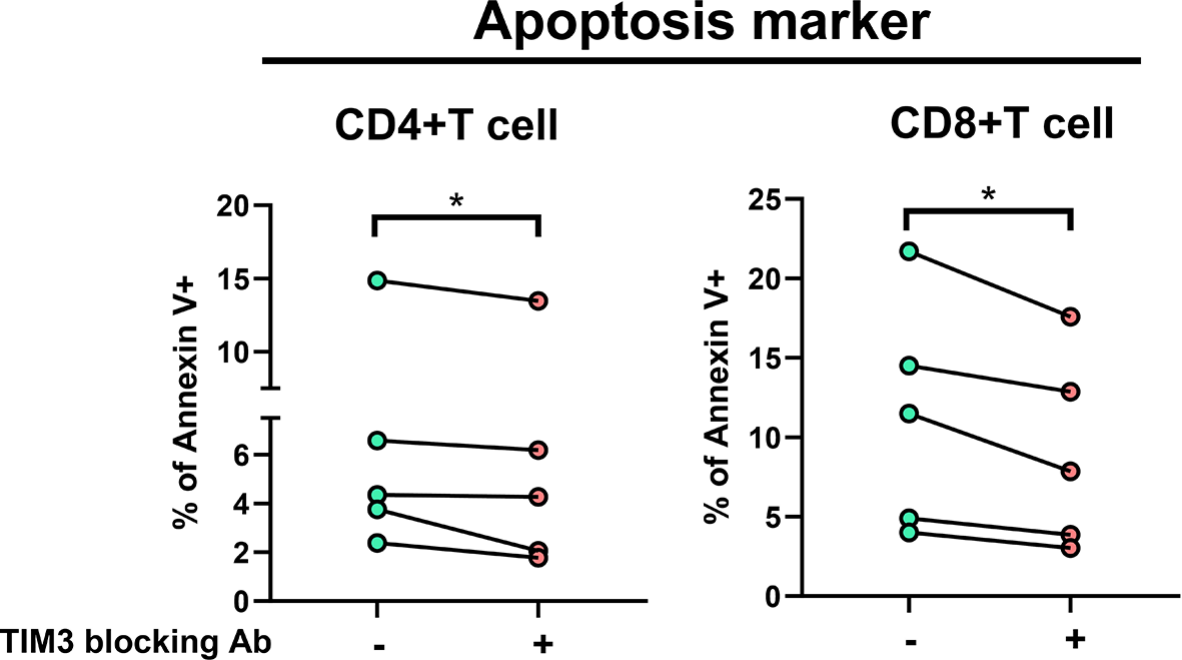
The proportions of Annexin V+ were measured under stimulation using heat-killed *Mycobacterium avium* (multiplicity of infection: 10) with or without TIM3 blocking antibody in PBMCs from five subjects (two controls and three patients with NTM-LD). Intra-subject changes are shown and were analyzed by the Wilcoxon test. One star indicates *p* < 0.05.

In TIM3 blocking assay in five subjects receiving additional MAC bacilli stimulation (**Figure 3**), total apoptosis % of CD4+ cells decreased from 6.4 ± 5.0% to 5.5 ± 4.8%, and that of CD8+ cells improved from 11.3 ± 7.3% to 9.0 ± 6.3% (both *p* < 0.05) after TIM3 blocking.

### The Cytokine Production According to TIM3 Level Under Stimulation Assay

**Figure 4:**
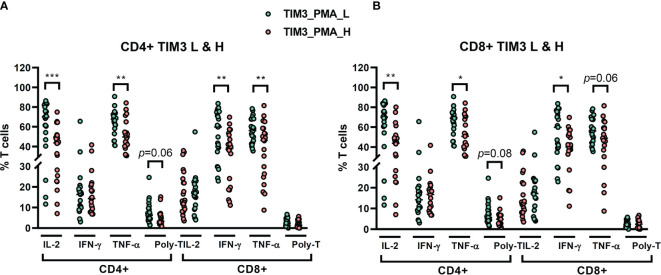
The percentages of cytokine production on CD4+ and CD8+ T cells from NTM-LD patients (*N* = 46) under stimulation with phorbol 12-myristate-13-acetate (PMA) plus ionomycin, plotted according to low or high levels of **(A)** CD4+ TIM3 and **(B)** CD8+TIM3, defined by the median levels. The cytokines, including IL-2, TNF-α, and IFN-γ, were measured by flow cytometry. Polyfunctional T cells were defined as positive for triple cytokines. The scatter plots are shown with cross bars of median levels. We analyzed the data with the unpaired *t* test. One star: *p* < 0.05; two stars: *p* < 0.01; and three stars: *p* < 0.001.

We recruited 46 NTM-LD patients for stimulation assay (29 MAC-LD, 12 MAB-LD, and 5 other NTM-LD). We measured cytokine production from T cells under PMA plus ionomycin by flow cytometry. We divided high and low TIM3 expression on CD4+ T cells in this assay by the median level of 1.48%, which was different from the cutoff value in the apoptosis results. The reasons were because the apoptosis experiment was according to baseline TIM3 expression (no stimulation) whereas the cytokine measurement was based on post-stimulation TIM3 expression. Although the PMA plus ionomycin stimulation induced high cytokine production, the TIM3 expression by contrast decreased. According to TIM3+ expression in PMA plus ionomycin stimulation, those with high TIM3+ expression on CD4+ had suppressed total IL-2+CD4+ (40.6 ± 18.3% vs. 62.9 ± 22.5%, *p* < 0.001), total TNF-α+CD4+ (52.3 ± 16.0% vs. 64.8 ± 12.9%, *p* = 0.006), triple-positive (IFN-γ, TNF-α, and IL-2) CD4+ T cells (5.0 ± 3.7% vs. 7.5 ± 5.3%, *p* = 0.063), IFN-γ+CD8+ (37.5 ± 17.1% vs. 54.3 ± 20.9%, *p* = 0.005), and TNF-α+CD8+ (42.2 ± 20.4% vs. 56.0 ± 13.0%, *p =* 0.008) T cells as compared with those with low TIM3+ expression on CD4+ cells (**Figure 4A**). According to high or low TIM3+ on CD8+, defined by a median level of 0.96%, the suppression patterns of cytokine production were similar to those of high or low TIM3+ on CD4+ cells (**Figure 4B**).

### Association Between Clinical Pattern and TIM3 Expression

**Figure 5:**
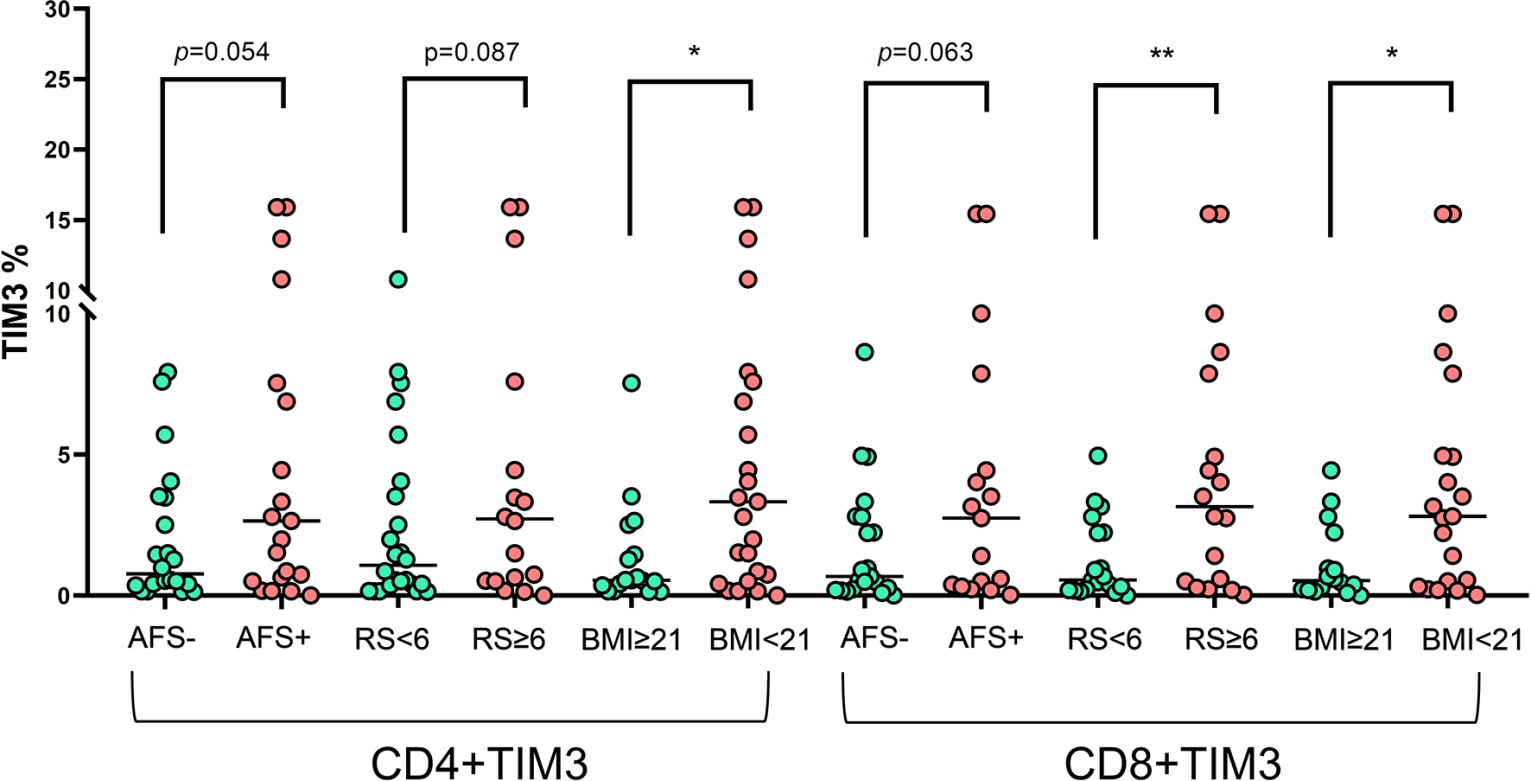
The expression of TIM3+ on CD4+ and CD8+ lymphocytes under PMA plus ionomycin according to acid-fast smear (AFS), body mass index (BMI), and radiographic score among the NTM-LD patients (*N* = 43). The scatter plots are shown with median levels. We analyzed the data using the unpaired *t* test. One star indicates *p* < 0.05; two stars mean *p* < 0.01.

In terms of clinical characteristics (**Figure 5**), patients with old age (≥65 years old) did not have different TIM3+CD4+ and TIM3CD8+ from those with age < 65 years old. Those with different genders also had similar TIM3+CD4+ and TIM3CD8+ expression. For bacilli burden, we classified the patients with NTM-LD whose PBMCs underwent *in vitro* PMA plus ionomycin stimulation into positive AFS (*n* = 22) or negative AFS (*n* = 21) subgroups and evaluated the between-group differences to understand the impact of NTM bacilli load on TIM3+ expression. The AFS-positive subgroup had higher TIM3+CD4+ (6.5 ± 10.3% vs. 2.0 ± 2.4%, *p* = 0.054) and TIM3+CD8+ (4.1 ± 5.1% vs. 1.8 ± 2.2%, *p* = 0.063) than the AFS-negative subgroup.

In addition, we classified the patients with NTM-LD into BMI < 21 kg/m^2^ (low) or ≥ 21 kg/m^2^ (high) subgroups for risk stratification of BMI. TIM3+CD4+ (6.2 ± 9.5% vs. 1.3 ± 1.9%, *p* = 0.044) and TIM3+CD8+ (4.2 ± 4.7% vs. 1.1 ± 1.3%, *p* = 0.015) were higher in the low BMI subgroup than in the high BMI subgroup. Patients with larger disease extent by radiographic score (>2 lung lobes or score > 6) had higher TIM3+CD4+ (2.5 ± 3.1% vs. 1.1 ± 1.3%, *p* = 0.087) and TIM3+CD8+ (6.6 ± 11.0% vs. 1.3 ± 1.4%, *p* = 0.008) than did those with smaller disease extent (≤2 lung lobes or score ≤6).

### TIM3 Expression After Anti-NTM Treatment

**Figure 6:**
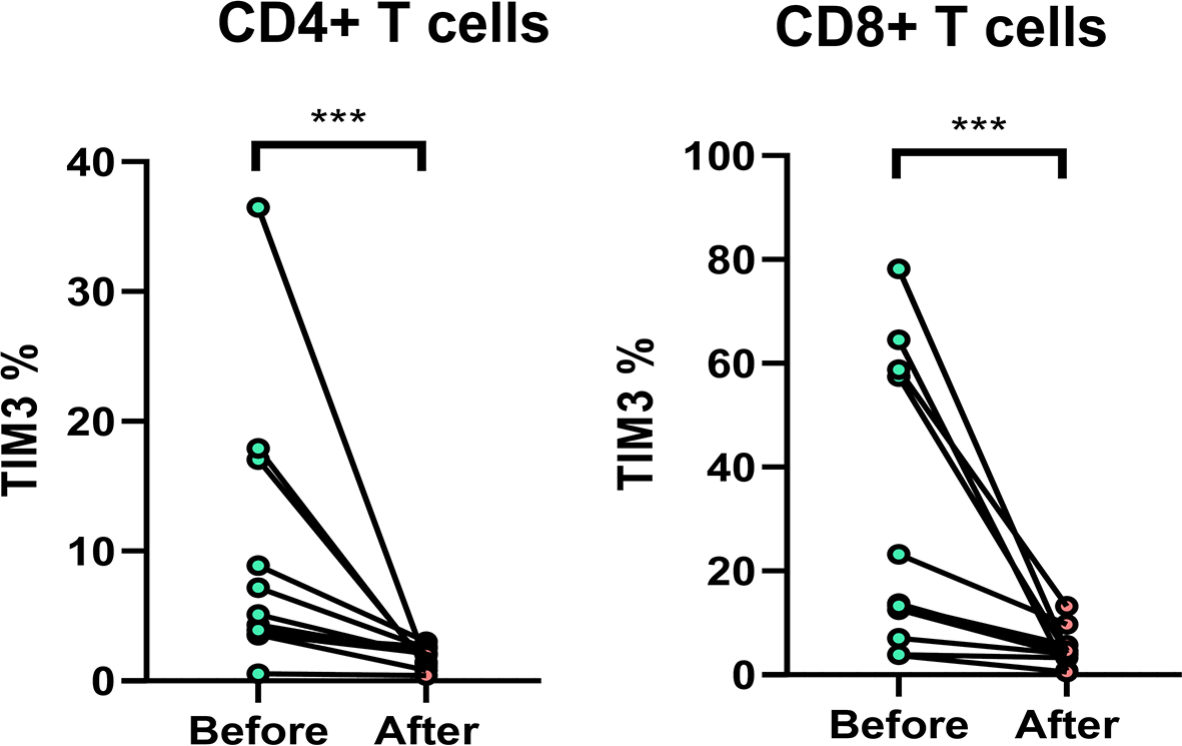
The expression of TIM3+ on CD4+ and CD8+ lymphocytes before and after anti-mycobacterial treatment. The intra-subject changes (before and after treatment) were compared using the Wilcoxon test. Three stars indicate *p* < 0.001.

Among the patients, 11 patients with anti-MAC treatment were followed up. The TIM3+CD4+ and TIM3+CD8+ on T cells decreased significantly after 2 months, from 9.7% ± 10.5% to 1.8 ± 0.8% (*p* = 0.002) and from 29.8% ± 28.7% to 4.8 ± 3.7% (*p* = 0.002), respectively (**Figure 6**).

## Discussion

The present study showed that NTM-LD patients had increased percentages of TIM3+CD4+ and TIM3+CD8+ lymphocytes regardless of age and gender. TIM3+ expression on T lymphocytes positively correlated with high Annexin V+ apoptosis. In PMA plus ionomycin stimulation, high TIM3+ expression was associated with low production of IL-2, TNF-α, and a lower percentage of triple-positive T cells on CD4+ cells and lower percentages of IFN-γ and TNFα-positive CD8+ lymphocytes. In terms of clinical characteristics, TIM3+ expression on CD4+ and CD8+ lymphocytes was higher in patients with higher bacilli burden, determined by a positive result for AFS; more disease extent, determined by radiographic score; and lower BMI than those of their counterpart groups, respectively.

TIM3 is located on the immune cell membrane and transduces inhibitory signals primarily on T cells, like other suppressive receptors such as PD-1 (12, 13). In viral infections, tuberculosis, or tumors (14–16), increasing expression of TIM3 on T cells is reportedly associated with cytokine reduction and poor prognosis (14, 17). However, the role of TIM3 has been rarely studied in NTM-LD. One Korean study showed that TIM-3-expressing T cells increased in MAC-LD patients in response to MAC stimulation and speculated that the increase might be related to attenuated cellular immunity (22). However, the mechanism leading from TIM3+ expression to immunity attenuation is unclear.

The present study also showed that TIM3+ over CD4+ and CD8+ both increased in patients with NTM-LD. After anti-NTM treatment, TIM3 expression significantly decreased, indicating that eradication of NTM might reduce TIM3 expression. At the initial stage of NTM-LD, TIM3 may have an anti-inflammatory effect. Patients with persistent NTM-LD might have subsequent TIM3 overexpression and T cell exhaustion, like the report of PD-1 in NTM-LD (10). The vicious cycle of NTM–TIM3 might be a possible pathogenesis of persistent infection of NTM-LD. However, such TIM3+ over-responsiveness is only hypothetical at present and will require future study to determine the responsible host pathogenesis.

For the possible role of TIM3 increment in NTM-LD, increasing apoptosis, inhibited proliferation, and suppression of cytokine production are the main ways that immune checkpoint receptors perform negative regulation of T cells (23, 24). Although the present study did not check lymphocyte proliferation status according to TIM3 expression, we confirmed that in NTM-LD, TIM3 expression was associated with increasing cellular apoptosis. In addition, blocking TIM3 could reverse cell apoptosis status significantly.

In regard to cytokine production from T cells, TIM3+ expression also demonstrated its role in cytokine attenuation. For CD4+ T cells, IL-2 and TNF-α were decreased significantly, as were polyfunctional T cells. IL-2 induces proliferation and differentiation (25). By contrast, TNF-α helps lymphoid tissue development and cellular differentiation to fight against intracellular pathogens (26). For CD8+ T lymphocytes, TNF-α and IFN-γ were attenuated in high expression of TIM3+ on T cells. IFN-γ upregulates pathogen recognition, processing, and activation of microbicidal effector functions (27). Overall, TIM3+-related decreased cytokine might lead to attenuation of CD4+-related proliferation and coordination, as well as CD8+-related cytotoxicity and pathogen defense.

In terms of clinical features, we found that TIM3 expression was higher in patients with high-grade AFS, indicating greater bacilli burden and severity of disease status. In cases of cellular impact by TIM3+ expression, TIM3+ might lead to decreased immunity, also known as exhaustion, which is more prevalent in patients with high AFS grade and severe NTM-LD. On the other hand, low BMI represents nutrition status and is correlated with low leptin (28), which is an upregulator for toll-like receptors (29) and a promotor of lymphocyte survival (30). Leptin deficiency might induce worse immunity upon NTM-LD and possible subsequent TIM3+ overexpression, but the details of the mechanism await further study.

Several limitations existed in this study. First, participants were enrolled at medical centers, so selection bias might exist, although patients with major underlying diseases were excluded. Second, the sample size was not large. Third, only TIM3, and no other immune checkpoints, was studied. Cross-linking between them should be studied in the future. Fourth, some analysis identified cross-sectional associations, but no causal relationships can be inferred. Determination of the direct mechanism may require future bench and animal studies. Last, the study was conducted in Taiwan, so generalization of the findings to other ethnicities and areas can be performed only after validation.

In conclusion, NTM-LD patients had higher expression of TIM3+ over CD4+ and CD8+ as compared with controls. The expression of TIM3 decreased after anti-NTM treatment. High TIM3+ might lead to cell exhaustion in NTM-LD through increased apoptosis and attenuated cytokine production. In addition, high TIM3 expression might result from a high bacilli burden, greater disease extent of NTM-LD, and lower BMI. Although many details are still understudied, we suggest that TIM3+ might be overexpressed in NTM-LD with T-cell exhaustion. Future study for TIM3 overexpression in NTM-LD is warranted.

## Data Availability Statement

The raw data supporting the conclusions of this article will be made available by the authors, without undue reservation.

## Ethics Statement

The studies involving human participants were reviewed and approved by the Research Ethics Committees of National Taiwan University Hospital (NTUH IRB No. 201407079RIND, 201505069RINC, 201701014RINC and 201705087RINA), Taipei Veterans General Hospital (IRB No. 2014-09-008BC) and Far Eastern Memorial Hospital (IRB: 107162-E,109004-E). The patients/participants provided their written informed consent to participate in this study. Written informed consent was obtained from the individual(s) for the publication of any potentially identifiable images or data included in this article.

## Author Contributions

C-CS and M-FW were involved in performing the experiment and collecting data. S-WP, C-CS, P-HW, S-LC, M-FW, and C-YH contributed to the data analysis and manuscript writing. C-CS was responsible for the study conceptualization and coordination. All authors contributed to the article and approved the submitted version.

## Funding

This study was supported in part by grants from National Taiwan University Hospital (NTUH. 109-S4552, 110-S5053, 110-T07), the Ministry of Science and Technology Taiwan (MOST 109-2628-B-075-026 and 109-2326-B-002-009-MY3), and the Far Eastern Memorial Hospital National Taiwan University Hospital Joint Research Program (108-FTN01 and 109-FTN04). The funders had no role in study design, data collection and analysis, decision to publish, or preparation of the manuscript.

## Conflict of Interest

The authors declare that the research was conducted in the absence of any commercial or financial relationships that could be construed as a potential conflict of interest.

## Publisher’s Note

All claims expressed in this article are solely those of the authors and do not necessarily represent those of their affiliated organizations, or those of the publisher, the editors and the reviewers. Any product that may be evaluated in this article, or claim that may be made by its manufacturer, is not guaranteed or endorsed by the publisher.
